# Emergency department utilization among recently released prisoners: a retrospective cohort study

**DOI:** 10.1186/1471-227X-13-16

**Published:** 2013-11-05

**Authors:** Joseph W Frank, Christina M Andrews, Traci C Green, Aaron M Samuels, T Tony Trinh, Peter D Friedmann

**Affiliations:** 1 Division of General Internal Medicine, Alpert Medical School of Brown University/Rhode Island Hospital, 111 Plain Street Building, Providence, RI 02903, USA

**Keywords:** Vulnerable populations, Mental health, Substance abuse, Emergency department

## Abstract

**Background:**

The population of ex-prisoners returning to their communities is large. Morbidity and mortality is increased during the period following release. Understanding utilization of emergency services by this population may inform interventions to reduce adverse outcomes. We examined Emergency Department utilization among a cohort of recently released prisoners.

**Methods:**

We linked Rhode Island Department of Corrections records with electronic health record data from a large hospital system from 2007 to 2009 to analyze emergency department utilization for mental health disorders, substance use disorders and ambulatory care sensitive conditions by ex-prisoners in the year after release from prison in comparison to the general population, controlling for patient- and community-level factors.

**Results:**

There were 333,369 total ED visits with 5,145 visits by a cohort of 1,434 ex-prisoners. In this group, 455 ex-prisoners had 3 or more visits within 1 year of release and 354 had a first ED visit within 1 month of release. ED visits by ex-prisoners were more likely to be made by men (85% vs. 48%, p < 0.001) and by blacks (26% vs. 16%, p < 0.001) compared to the Rhode Island general population. Ex-prisoners were more likely to have an ED visit for a mental health disorder (6% vs. 4%, p < 0.001) or substance use disorder (16%vs. 4%, p < 0.001). After controlling for patient- and community-level factors, ex-prisoner visits were significantly more likely to be for mental health disorders (OR 1.43; 95% CI 1.27-1.61), substance use disorders (OR 1.93; 95% CI 1.77-2.11) and ambulatory care sensitive conditions (OR 1.09; 95% CI 1.00-1.18).

**Conclusions:**

ED visits by ex-prisoners were significantly more likely due to three conditions optimally managed in outpatient settings. Future work should determine whether greater access to outpatient services after release from prison reduces ex-prisoners’ utilization of emergency services.

## Background

Over 7 million adults in the U.S. were under correctional supervision in 2009, and more than half a million leave prison and return to their communities each year [1,2]. Ex-prisoners suffer from increased rates of many chronic medical conditions, including mental illness and diseases of addiction [3-9]. The risks faced upon community re-entry make this period particularly dangerous. Mortality is increased substantially [10-12]. Substance use, accidental drug overdose and suicide play significant roles [13-19].

Poor access to health services during the period post-release, specifically for substance use and mental health disorders, may contribute to poor health outcomes. Disparities in access to ambulatory medical care as well as more specialized services such as HIV care exist [20-23]. Increased disease prevalence and poor access may lead to increased utilization of acute care services such as emergency department (ED) services, particularly for substance use and mental health disorders. Such utilization may lead to poor continuity of care for patients and contribute to overcrowding and increased costs for hospitals. However, patterns of acute care utilization by ex-prisoner populations are not well understood.

A study by McCorkel demonstrated rates of hospital discharge among ex-prisoners with a history of drug abuse that were more than three times that of a comparable national sample [24]. Work by Freudenberg found the rates of health service utilization after release from prison increased in a female cohort but decreased among adolescent males compared to utilization prior to incarceration [25]. Finally, a study by Hiller et al. found that rates of ED and hospital use were increased in male prisoners with co-occurring mental health and substance use disorders [26]. These studies relied on self-report and focused on subgroups within the larger incarcerated population, limiting internal and external validity. To date, ED utilization of ex-prisoners has not been studied using external measures of utilization nor has it been compared to the general population.

Therefore, we sought to describe patterns of ED utilization by a cohort of recently released prisoners in Rhode Island. We also sought to compare proportions of ED utilization for mental health disorders, substance use disorders and ambulatory care sensitive conditions (ACSC) among the ex-prisoner population compared to that of the general population.

## Methods

### Study protocol/data sources

We merged data from several sources for the present study. First, the Rhode Island Department of Corrections (RIDOC) provided data for 6,046 sentenced adults released from state correctional facilities between January 1, 2007 and December 31, 2008 (“Dataset A”). These data included demographic data, admission and release dates and ZIP code of residence for each individual. The Rhode Island Department of Corrections is unique in that it operates a unified correctional system. All sentenced individuals are housed in 1 of 7 facilities located on a single campus that is located approximately 6 miles from the state’s urban center and its academic medical center. RIDOC housed approximately 3900 individuals in 2008, and 77% of released individuals returned to the counties served by study hospitals [27].

RIDOC data was linked to the electronic health record of a major hospital system in Rhode Island (“Dataset B”). The system’s three hospitals include the state’s urban, tertiary care hospital (“Hospital B”) and together are responsible for approximately 50% of ED visits in the state [28]. We identified all ED visits occurring within 1 year of each ex-prisoner’s first release during the study period. Data included intake, service and discharge records. Data were linked using first name, last name and date of birth. A research analyst with extensive experience working with electronic health record data performed data linkage and extraction electronically. These data were de-identified once this linkage was made.

To obtain data on visits by the Rhode Island general population, the Rhode Island Department of Health (RIDOH) provided data on all ED visits in the hospital system from January 1, 2007 to December 31, 2009 (“Dataset C”). Data included patient age, gender, race, ethnicity, residence, diagnosis (ICD-9), year of visit, treatment facility and ZIP code of residence. No unique identifiers were included in these data and therefore visits could not be linked to individuals across facilities or over time. We obtained data on population size and unemployment rates from the 2000 United States Census (“Dataset D”). We linked census data with ex-prisoner and general population visit data using ZIP codes. We excluded visits by individuals outside of Rhode Island and nearby Bristol County, MA as they were deemed unlikely to access the hospital system of interest.

Finally, we combined visit-level data from datasets A, B, C and D to create the final sample, which included 333,369 ED visits.

### Study measures

We created three dependent variables at the level of the ED visit, indicating whether each visit had a primary diagnosis of one of three types of diagnosis. For the first dependent variable, we measured whether a visit had a primary diagnosis of a mental health disorder. To create this variable, we used the New York University Emergency Department (NYU ED) Algorithm, which uses International Classification of Diseases, Clinical Modification, Ninth Revision (ICD-9-CM) codes to classify ED visits into categories [29]. We created a dichotomous variable in which visits categorized as mental health-related by this algorithm were coded affirmatively. For the second dependent variable, we measured whether a visit had a primary diagnosis of a substance use disorder. Two of the NYU ED algorithm categories were used to create this variable: alcohol and other substance use-related visits. We coded visits affirmatively if the algorithm indicated that a visit was related to alcohol or other substance use. For the third dependent variable, we measured whether a visit had a primary diagnosis of an ambulatory care sensitive condition [30]. These conditions include several common physical health-related conditions such as asthma, hypertension, and diabetes. We coded visits with an ICD-9-CM code indicating a primary diagnosis of an ambulatory care sensitive condition affirmatively.

The study’s independent variable was ex-prisoner status, defined as an index release from the state’s correctional facility within the year prior to the ED visit. In these analyses, we do not differentiate between those visits occurring while an individual was living in the community and visits occurring while re-incarcerated during the year following the index release.

### Study covariates

At the individual-level, we included variables for age (measured as a continuous variable), gender, race/ethnicity (black, Hispanic, white, other race), and the hospital facility in which the visit occurred. We excluded visits by individuals under 18 and over 70 years of age from the general population sample to ensure appropriate comparison with the ex-prisoner sample, which did not include children and included few older adults. Indicator variables for year controlled for changes in ED visitation patterns over time. At the ZIP code-level, we measured unemployment rate (measured as tenths of a percentage point) as a surrogate measure of both economic disadvantage and rate of uninsurance. Finally, we measured community population at the ZIP code-level. As these population data were highly positively skewed, a natural logarithmic transformation was performed to decrease the influence of extreme values.

### Data analysis

We first performed descriptive statistics within the ex-prisoner cohort (N = 1434). We determined the timing of first ED visit after release, both overall and for the three diagnosis types of interest. We examined the relationship between first release from prison and first ED visit and used the chi-square test to assess associations between the timing of first ED visits and several relevant individual-level characteristics. We next compared visits by the ex-prisoner and general populations across several patient- and community-level characteristics. We used the chi-square test for differences in categorical variables and analysis of variance (ANOVA) for differences in continuous variables. We used Satterthwaite corrected *t*-tests to account for highly unbalanced variances across the two groups due to the large difference in the number of ED visits among the ex-prisoner and general populations.

We created random effects logistic regression models to examine the association between ex-prisoner status and the proportion of ED visits within ex-prisoner and general population groups for three outcome conditions. We assumed a logistic distribution with a logit-link function. To account for potential correlation among individuals living in the same community, we assumed an exchangeable covariance structure among patients from the same ZIP code. We created three separate models to investigate the relationship between ex-prisoner status and each of the three outcomes of interest: mental health-related visits, substance use-related visits and ambulatory care sensitive condition-related visits. We adjusted for patient gender, race/ethnicity, age, visit year, visit facility at the individual-level as well as unemployment rate and total population at the level of the ZIP code. We explored interactions between the independent variable, ex-prisoner status, and patient age, gender and race/ethnicity. We found no significant interactions and so did not include these terms in the final models.

We report results as odds ratios with 95% confidence intervals. We performed all statistical analyses using SAS version 9.3 and STATA MP version 11. The study was approved by the Miriam Hospital Institutional Review Board and by the Rhode Island Department of Corrections Medical Research Advisory Group.

## Results

### Description of ex-prisoner cohort

Among 6,046 individual ex-prisoners released during the study period, 1,434 (23.7%) had at least 1 ED visit within the state’s largest hospital system within 1 year of release. This group had a mean age of 34.5 years (SD 10.1), was predominantly male (86.7%) and the majority were white (53.9%). The median length of incarceration prior to first release during the study period was 188 days (IQR 54–288 days) with 263 individuals (18.3%) incarcerated longer than 1 year. Nearly 1 in 4 individuals were re-incarcerated at least once during the study period (N = 338/1434; 23.6%). The median time to re-incarceration during the first year after release was 122 days (IQR 56–203 days) and these individuals spent an average of 158 days (SD 97) in the community during this year.

### Description of ex-prisoner visits

**Figure 1:**
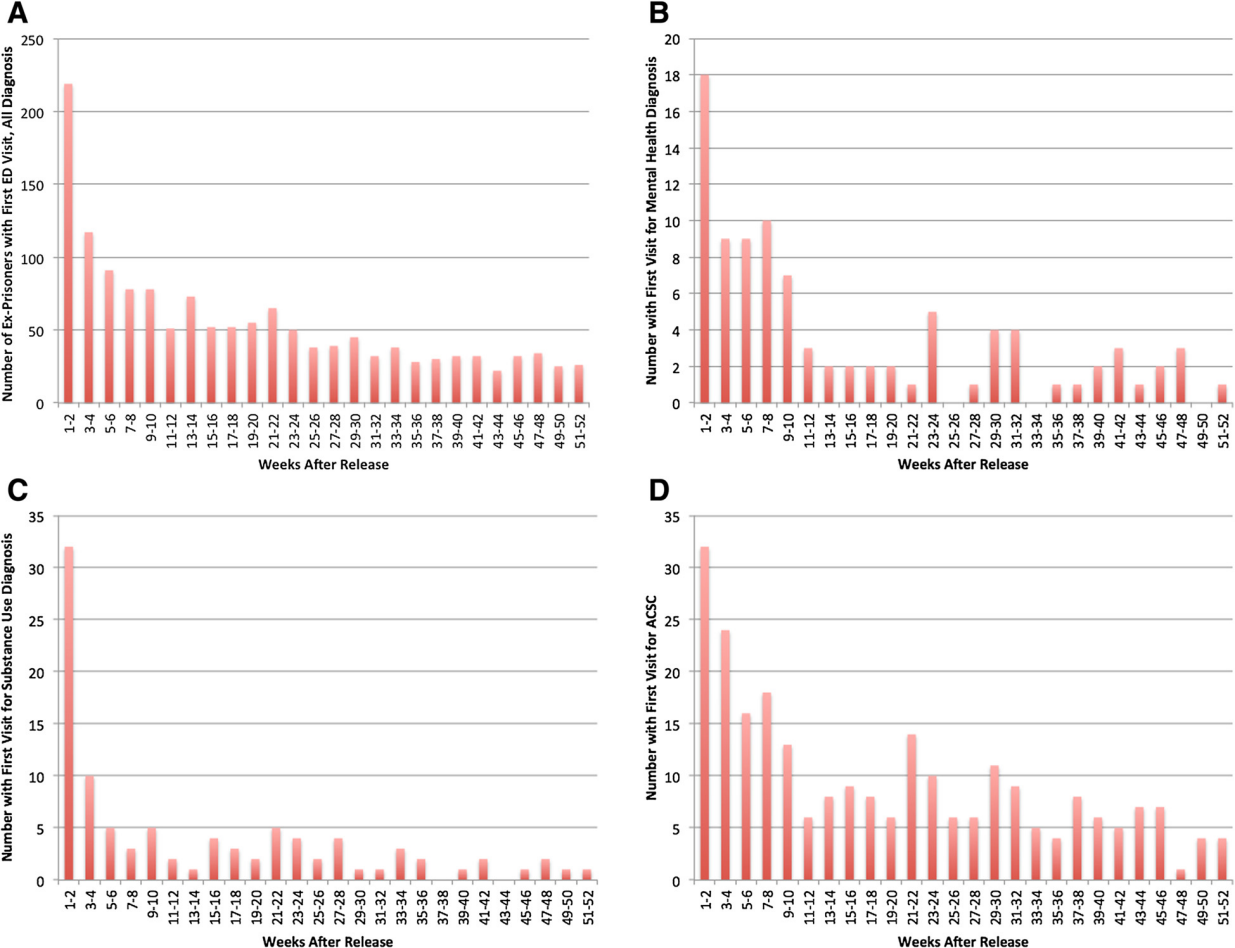
**Timing of first emergency department visit.** After index release from prison for **A)** any diagnosis; **B)** Mental health diagnoses; **C)** Substance use diagnoses; **D)** Ambulatory care sensitive conditions (ACSC).

The ex-prisoner cohort accounted for a total of 5,145 ED visits within 1 year of release from prison, an average of 3.6 visits per person. Within this group, 455 individuals (31.7%) had 3 or more ED visits and 102 (7.1%) had 10 or more ED visits. A single individual in the ex-prisoner cohort accounted for 114 ED visits in the year following release. The first visit following release from prison occurred within the first 2 weeks for 219 individuals (15.3%), within the first month for 354 individuals (24.7%) and within the first 12 weeks for 634 individuals (44.2%) (Figure 1a). The rate of first ED visits was 2.6 times greater during the first 2 weeks than during weeks 3–12. This pattern was present for first visits for each of three diagnosis types: mental health disorders (2.4×), substance use disorders (6.4×) and ambulatory care sensitive conditions (2.1×) (Figure 1b- 1d). Finally, age ≥ 45 years (28% vs. 12%; *P* < .001), white race (20% vs. 10%; *P* < .001) and subsequent re-incarceration (19% vs. 14%; *P* = .04) were significantly associated with ED visit within the first 2 two weeks after release.

### Diagnosis-specific comparison to general population visits

At the level of the ED visit, we compared 5,145 visits by ex-prisoners and 328,224 visits by members of the general population (from a total of 1,048,319 individuals over three years). Compared to the general population (Table 1), ED visits by ex-prisoners were less likely to be made by women (15% vs. 53%) and more likely to be made by black individuals (26% vs. 16%). There were no differences between the two groups with regard to age. Demographic characteristics of visits by ex-prisoners closely reflect the composition of the ex-prisoner population. In unadjusted analyses, ex-prisoners’ visits were more likely to be due to mental health disorders (6% vs. 4%) and substance use disorders (16% vs. 4%) than visits by the general population. However, visits by ex-prisoners were no more likely to be due to ambulatory care sensitive conditions than visits by the general population (13.8% vs. 13.6%).

**Table 1:** Characteristics of emergency department visits by ex-prisoner and general population groups in the state of Rhode Island, 2007-2009

	**Ex-prisoner visits**	**General population visits**	***P***
**Total Visits, N**	5,145	328,224	
**Mean age at time of visit, years (SD)**	38.7 (10.6)	38.7 (14.1)	0.91
**Female, N (%)**	776 (15.1%)	170,841 (52.1%)	<.001
**Race/ethnicity, N (%)**			
**Black**	1311 (25.5%)	51,925 (15.8%)	<.001
**Hispanic**	454 (8.8%)	51,958 (15.8%)	<.001
**White**	3312 (64.4%)	215,381 (65.6%)	<.001
**Other Race**	68 (1.3%)	10,930 (3.3%)	<.001
**Mental health visits, N (%)**	328 (6.4%)	12,799 (3.9%)	<.001
**Substance use visits, N (%)**	808 (15.7%)	13,799 (4.2%)	<.001
**ACSC visits, N (%)**	704 (13.8%)	44,662 (13.6%)	0.79

*Note.* ACSC ambulatory care sensitive condition.

Ex-prisoner status was significantly related to all outcomes of interest in random effects logistic regression models (Table 2). Visits by ex-prisoners were 43% more likely to be due to a mental health disorder (AOR 1.43; 95% CI 1.27-1.61). Conversely, visits by members of racial/ethnic minority groups were less likely to be due to a mental health disorder. Age was negatively associated with a visit being related to a mental health disorder while gender showed no significant association with the likelihood of a visit being due to a mental health disorder.

**Table 2:** Multivariable-adjusted odds ratios for emergency department visits related to mental health disorders, substance use disorders or ambulatory care sensitive conditions (ACSC)

	**Mental health**	**Substance use**	**ACSC**
	**Adj. OR (95% CI)**	**Adj. OR (95% CI)**	**Adj. OR (95% CI)**
**Ex-prisoner**	1.43 (1.27-1.61)	1.93 (1.77-2.11)	1.08 (1.00-1.18)
**Age (years)**	0.99 (0.99-0.99)	1.03 (1.02-1.03)	0.99 (0.99-0.99)
**Female**	0.99 (0.95-1.02)	0.31 (0.29-0.32)	1.28 (1.25-1.31)
**Race/ethnicity**			
White	1 [Reference]	1 [Reference]	1 [Reference]
Black	0.68 (0.64-0.72)	0.55 (0.52-0.58)	1.26 (1.22-1.29)
Hispanic	0.69 (0.65-0.73)	0.45 (0.43-0.48)	1.00 (0.97-1.03)
Other race	0.70 (0.62-0.78)	0.42 (0.37-0.50)	0.95 (0.89-1.00)
**Visit location**			
Hospital A	1 [Reference]	1 [Reference]	1 [Reference]
Hospital B	2.26 (2.02-2.51)	1.86 (1.65-2.10)	0.92 (0.86-0.98)
Hospital C	1.10 (0.98-1.24)	0.40 (0.35-0.46)	1.09 (1.02-1.16)
**Visit year**			
2007	1 [Reference]	1 [Reference]	1 [Reference]
2008	1.06 (1.02-1.11)	0.93 (0.89-0.97)	1.09 (1.07-1.12)
2009	1.11 (1.06-1.16)	0.93 (0.89-.097)	1.06 (1.03-1.08)
**Unemployment rate**	4.24 (1.96-9.16)	1.33 (0.88-2.02)	1.94 (1.43-2.65)
**Zip code population (log)**	1.07 (0.95-1.21)	1.01 (0.94-1.09)	1.07 (1.01-1.13)

*Note. OR* odds ratio, *CI* confidence interval, *ACSC* ambulatory care sensitive condition. Estimates were calculated with the use of random effects logistic regression models. All covariates listed were included in models.

Visits by ex-prisoners were nearly twice as likely to be due to a substance use disorder (AOR 1.93; 95% CI 1.77-2.11). Age was also associated with a higher likelihood of a visit being substance use-related while visits by women and members of racial/ethnic minority groups were less likely to be due to substance use.

Finally, ex-prisoner status was associated with having an ED visit due to an ambulatory care sensitive condition, though the effect size was small (AOR 1.09; 95% CI 1.00-1.18). While the descriptive comparison found no statistically significant difference, after adjusting for individual and community-level characteristics, visits by ex-prisoners were 9% more likely to be due to an ambulatory care sensitive condition. Visits by women and blacks were also more likely to be due to an ambulatory care sensitive condition.

## Discussion

In this study, we found that early ED utilization following release from prison is common among a cohort of ex-prisoners in the state of Rhode Island and is associated with older age, white race and subsequent re-incarceration. Additionally, by comparing ED visits by ex-prisoners to those made by the state’s general population, we found that visits by ex-prisoners were more likely to be related to mental health disorders, substance use disorders and ambulatory care sensitive conditions than were visits by Rhode Island residents of the same age, sex, race and location of residence. While incarceration disproportionately afflicts poor young males from racial/ethnic minority groups, our findings demonstrate an association between recent release from prison and condition-specific utilization of the ED after controlling for these factors.

The ex-prisoner population in our study reflects demographic patterns seen in incarcerated populations nationally. Men, especially members of racial/ethnic minority groups, are disproportionately represented. A majority of ex-prisoners return to major metropolitan areas both in Rhode Island and nationally. As the catchment areas of the hospitals studied include Rhode Island’s urban areas, we believe the utilization captured in this study is representative of a majority of the state’s ex-prisoner population.

The three types of ED utilization examined in this study share in common the fact that each is optimally managed in a community-based, longitudinal manner rather than episodically in emergency and inpatient settings. A plausible common pathway for increased ED utilization is one of poor access to care in the community in the period following release from prison, particularly given the high rates of early ED utilization following release seen in this cohort. The increased likelihood of ED visits due to these conditions among ex-prisoners is consistent with previous work demonstrating disparities in access to care by race, income level and insurance status [31-33]. Each of these characteristics is over-represented in the ex-prisoner population. However, recent release from prison appears to be independently related to likelihood of ED visit being related to mental health disorders, substance use disorders and ambulatory care sensitive conditions.

An increased likelihood of ED visits being related to mental health and substance use disorders is consistent with work by Hiller and colleagues demonstrating increased health services utilization by prisoners with co-occurring mental health and substance use disorders compared to prisoners with only one or neither of these conditions [26]. Additionally, the increased likelihood of ED utilization for ambulatory care sensitive conditions by the ex-prisoner cohort was small but statistically significant. This latter finding is consistent with work by Kulkarni et al. showing unmet needs for medical and dental care among ex-prisoners [20]. This study complements these survey data with the use of electronic health record documentation from a large hospital system as well as by the context provided by the general population comparison group.

The patterns of ED utilization by ex-prisoners shown in this study are particularly problematic in light of prior research demonstrating increased mortality following release from prison. Among former inmates in Washington state, Binswanger et al. showed that drug overdose was the leading cause of death in the year following release with a relative risk of 12.2 compared to the general population [10]. Rates of death due to homicide, liver disease, suicide and motor vehicle accidents were more than three times that of the comparison group. The finding of increased risk of death by suicide and drug overdose has supported by multiple studies [13-19]. Each of these outcomes is plausibly associated with mental health and/or substance use disorders.

Our findings add to this body of knowledge by characterizing a predictable yet preventable complication of these diseases in the form of ED utilization. Similarities between documented patterns of mortality in ex-prisoners and the ED utilization seen in this study suggest these data may capture different points along the same disease trajectory, reflecting a real need for medical care and rational response to poor access. They also reinforce a need for evidence-based interventions to provide coordinated care during community re-entry, particularly for those ex-prisoners with mental health or substance use disorders. While existing interventions show promise, their impact on clinical outcomes and health service utilization requires further investigation [34,35].

Finally, study findings demonstrate significant differences in condition-specific ED utilization by gender and race/ethnicity within the ex-prisoner cohort. The underlying mechanisms cannot be adequately addressed with these data. The effect of criminal justice involvement on health disparities in general requires further study [36]. Studies suggesting the potential for incarceration to attenuate disparities in chronic disease outcomes and access to care highlight the challenges facing researchers seeking to understand the complex interplay between incarceration and the many other social determinants of health [11,37].

These findings are timely for several reasons. First, the Affordable Care Act (ACA) will extend insurance coverage eligibility to millions of low-income adults, dramatically altering access to coverage among the population leaving correctional facilities [38]. Second, specific ACA provisions are expected to improve access and fragmentation within the nation’s mental health and substance use disorder treatment systems [39]. The healthcare utilization seen in this study suggests that the nation’s criminal justice system offers a high-yield point of contact for the state and federal agencies responsible for enrolling newly eligible individuals in coverage. Such targeted enrollment could facilitate access to the ambulatory care among this high-risk group, potentially impacting need for ED services and thereby ED overcrowding. Additionally, future work should seek to determine the impact of recent legislation on access, utilization and health outcomes among the nation’s criminal justice populations.

This study should be interpreted in the context of its limitations. ED utilization was limited to visits occurring in a single hospital network in the state of Rhode Island and may not be generalizable to other settings. Our study did not capture visits at other hospitals in Rhode Island or in neighboring states nor does it account for visits to community-based sites such as urgent care centers. Lacking these data likely would have underestimated the number of ED visits by the study population. Thus, we can be confident that the observed associations are as high or higher than estimated.

Data limitations resulting from de-identification of ex-prisoner data as well as those inherent in data from the Rhode Island Department of Health prevented linkage of visits by specific individuals over time or between hospitals. Our main analyses therefore compare diagnosis-specific proportions of health care utilization of Rhode Island’s ex-prisoner population to that of the state’s general population. Further study should seek to better characterize rates of utilization over time by ex-prisoner populations.

The description of the case mix in our study is limited by its reliance on ICD-9 coding and absence of a more detailed chart review. As only primary ICD-9 codes were used, we are unable to identify those ED visits in which diagnoses of interest were not made or documented by the treating physician. Thus, the true impact of mental health disorders, substance use disorders and ambulatory care sensitive conditions is likely underestimated. Finally, we were unable to identify the ex-prisoner population within the Department of Health dataset. Due to the differences in size between the two groups, any impact on our findings would be minimal and would bias results toward the null.

## Conclusions

Emergency department visits by ex-prisoners within 1 year of release from prison were significantly more likely to be related to mental health disorders, substance use disorders and ambulatory care sensitive conditions. Each of these conditions is optimally managed with community-based, ambulatory services. Future work should determine whether greater access to outpatient mental health, substance use and primary care services during the transition from prison might reduce ex-prisoners’ need for and utilization of emergency services.

## Competing interests

The authors declare that they have no competing interests.

## Authors’ contributions

All authors have made substantial contributions to conception and design, acquisition of data, and analysis and interpretation of data. JWF and CMA drafted the manuscript. TCG, AMS, TTT and PDF were involved in revising the manuscript critically for important intellectual content. All authors read and approved the final manuscript.

## Pre-publication history

The pre-publication history for this paper can be accessed here:

http://www.biomedcentral.com/1471-227X/13/16/prepub
